# Supplementary material for: Patterns of Intron Gain and Loss in Fungi
Source: PLoS Biol. 2004 Nov 30;2(12):e422. doi: 10.1371/journal.pbio.0020422 (PMC532390; doi:10.1371/journal.pbio.0020422)
Supplement: Table S1 — Also available at http://genes.mit.edu/NielsenEtAl/. (4.3 MB ZIP). [file pbio.0020422.st001.zip › NielsenEtAl/html/658.html]

AN8812.1.NCU04021.1.MG00937.1.FG08373.1


```
 CLUSTAL W (1.82) Multiple Sequence Alignments - Introns Inserted


Sequence 1: NCU04021.1	1644 aa
Sequence 2: MG00937.1	1654 aa
Sequence 3: FG08373.1	1624 aa
Sequence 4: AN8812.1	1626 aa
Alignment Length: 1751 aa
Number Identitical Residues: 516 aa
Alignment Score (without introns) 29287


MG00937.1 	-MAFIRQTITL~VKKNFLIILWRHSTATVLRAFLLPIFLAAFLSFARFFFVPP-------
NCU04021.1	-MAIFSQTWTL~TRKNLLIVLNRHTNATIIRAFVLPVLVVAFLSFAKNLFVPY-------
FG08373.1 	-MAFIKQVGTL~MGKNFRILLFRHLALCIWMAFILPIFLAALFSFTKNLLVPP-------
AN8812.1  	MTVPMLGRYSY1VDSDSQKPTGCPCSPSTLDRFSSIDPACALCGIHVCTSKPAPWHSLRC
          	  . :    :    .:                *       *: .:      * .  :  .

MG00937.1 	-------A~RNGVADSLAIRSLGDSLRVAGETSRNTVVFVDGGHKTGDIDRVINNLTSQV
NCU04021.1	-------A~VFGISKVHPVRSLSDGLQASSGTGRNTVAFVNNGLRGGEIDRVIQELATVV
FG08373.1 	-------A~KFGIGNVAPLMPLDEAITKATNNGREKLVLVNNGNNDTDVNRVFSSVKSKF
AN8812.1  	MSDVNADP1TYGIGEPTRVQSFSNALESVG-AGRNRLIFVDNGFVGGSIEQVINEVAEPA
          	 :. .:..   *:..   : .:.:.:      .*: : :*:.*    .:::*:..:    

MG00937.1 	EDAGKT------VFRLDRENEIQRVCKSSLRGVSQCYGAAVFN~GSPD--FGGIWNYTLR
NCU04021.1	RDAGKN------ASIAESEQDLIWTCRASLRGVTPCYGAVVFY~SSPDEGSGGIWNYTLR
FG08373.1 	EDAAKSAEVDFEVVFVDNEDELTNNCPSNLRGVSRCFGAIVME~SSSQ----GIWNYTIR
AN8812.1  	RTSQYE------VHVLSDESEMRELCRTSLR-----------E1GPAE-----AWNYTIR
          	. :         .   . *.::   * :.**             ...:      ****:*

MG00937.1 	MDGALGPLGRIDVESNTRDAQIFVLPFQRAVDAAITGLNSSSNSSPL-SN~SHEYVFTSL
NCU04021.1	ADSALG-TGKINVDKDDNDAQVYMLPLQRAVDAAIT---RNGQQKLP-EE~QDEYPFTSL
FG08373.1 	ADGAFEYAPAKFRVDKANDQQIYIMPLQHAVDQAITR-LNNSNSTTL-DD~ISEYPFTSM
AN8812.1  	TDSVLG--KGIDVGNSNNDQQVYLLPFQRSIDWAIARTNSSFDASTLPEE0TLEYPYTSL
          	 *..:         .. .* *::::*:*:::* **:    . : .  ..:   ** :**:

MG00937.1 	TQSERDEEIRVRYQQAVTNFMGVAFILAIIGVCYHLTGFMATEREIGMSTLIESMMPTRQ
NCU04021.1	TKEERADRVRQLYQKTIYNVLGVTFLISVIGVCYHLVGFMASERETGMSTLVEAMMFTKR
FG08373.1 	TPEERDREIRRRFQKAIIGWMGVAFLGTVVWVTYHLTGFIATERESGMSTLIDAMMPVRK
AN8812.1  	NEEGRKDRIRTRYMGAIIDVIAVAVFIGMVGVTYQLTGLIAMERELGMSQLIDCMMPDSS
          	. . *  .:*  :  :: . :.*:.:  :: * *:*.*::* *** *** *::.**    

MG00937.1 	RWQAQAARLLSYHIAFDVIYLPGWIIGALILQLGVFRHTSTAMYVFYNITAGLALSSMSI
NCU04021.1	TWEGQAARLLSYHLGFTLLYLPGYIVSSIIMWAAVFRTSSVGILIIYHILAGLSLASFSI
FG08373.1 	PWLAMVARIIAHHLSFSLIYLPAWVIGSVIVRGGVFAKTSMAIVLVFHVIAGLAFSSISL
AN8812.1  	PWMSQAARFCAAHLALDIVYGPGWIIMGAILKAGVYSETSAGITVVYHILCGLALSSFSI
          	 * . .**: : *:.: ::* *.::: . *:  .*:  :* .: :.::: .**:::*:*:

MG00937.1 	FGAAFFKKAQLSGVVVTIVYCVLAIVAQAISYPSTAQVTILSLLFAPCNYVYFIANTARW
NCU04021.1	LGAAFFKKAQLSGISIVILYILLGVIAQIITAPKTATVVVLSLLFMPCNYTYFIAYLARF
FG08373.1 	LFASIFKKAQLSGITAILVVAILGIVSQVLTSPGTVPVTVLSFLFTPSAFVFFIADMAHF
AN8812.1  	FGASFFRKAQLSGISVVLACLLLGVVAQMAPAKSNGPVVILGLLFPSMNYVYFSIFVARW
          	: *::*:******:   :   :*.:::*  .   .  *.:*.:** .  :.:*    *::

MG00937.1 	EAKKWPTDLTDVPPNSAWDIPAIWLWVFLIIQIIVYPIVGALIERYLYGTATKGRNIVNG
NCU04021.1	EAKDMATNLGKSAPDSPSQVAGIVFWIFLIVQIFAYPILGAIIERYLHGTTSSTRNIAYG
FG08373.1 	EEVERPTNLFESAPNSPSALPGIVLWVFLIIQIFAYPLIGAFIEHALFSNDTPGRKILVN
AN8812.1  	ERENSAAILTESAPNNPWSLPGVVLWVLLILQIIIYPMLAAVVERMLYGTASKNRHVSSS
          	*  . .: * . .*:..  :..: :*::**:**: **::.*.:*: *... :  *::  .

MG00937.1 	---GEMGGEAAVRLEGFTKIYKPGFIRRMFS-FISKPKPAVRAVDGLTLSAGRGQILALL
NCU04021.1	E-NSELGPDNAVQLDGFTKIYKPGPFRNMFS-FLSKPREPVIAVNGLTLTARRGQILVLL
FG08373.1 	QSDVDRMGSNAVQLRQFSKTYNPNFFSRLFR-RGANKKQPVRAVSELDLNIGQGQIIALL
AN8812.1  	------GTSAALSIRNLNKIYRPGWFYRTIGPLFGSNRQTVHAVNNLSIDVNKGQIMVLL
          	        . *: :  :.* *.*. : . : .  .. : .* **. * :   :***:.**

MG00937.1 	GANGSGKSTSLDAIAGINRLTSGSITIDG---TGGLGIAPQKNVLWDDVTVEEHIRIFNS
NCU04021.1	GANGSGKSTTLDIIAGINKLSSGSITVDG---TGGLGIAPQKNVLWDELTVEEHLRIFNR
FG08373.1 	GANGSGKSTTLDAISGMNKLTSGSIEIDG---RGGLGIAPQKNVIWDDLTVEEHIRIFNH
AN8812.1  	GANGSGKSTTLDAIAGLTKLSSGDININYGTEEPGFGLCPQKNVLWDNLTVKEHVKIFNR
          	*********:** *:*:.:*:**.* :: .:.  *:*:.*****:**::**:**::*** 

MG00937.1 	LKAPANKSSKEEIRQLVEAVDLTKKIKARSKTLSGGQKRKLQLGMMLTGGSAVCCVDEVS
NCU04021.1	LKSPNQLATKEDILELISGVDLAQKVDARSGTLSGGQKRKLQLGMMLTGGSAVCCIDEVS
FG08373.1 	LKAPNDRADAHQIEELIRSIDLYAKRNAFAKTLSGGQKRKLQLGLMLTGGSAVCCVDEVS
AN8812.1  	LKSTGKVDTEEQIMHLLRDCDLQKKAKARSKTLSGGQKRKVQLAMMFTGGSPICCVDEVS
          	**:. .    .:* .*:   **  * .* : *********:**.:*:****.:**:****

MG00937.1 	SGLDPLSRRKIWDILLAERGKRTMILTTHFLDEADLLADHIAVLSKGTLRAEGSSVELKN
NCU04021.1	SGIDPLSRRKIWDILLAERGHRTMILTTHFLDEADLLADHIAIMSKGTLRAEGSSVELKN
FG08373.1 	SGIDPLSRRKLWDIILAERGRRTMILTTHFLDEADLLADYIAILSKGTLRAEGSSVELKD
AN8812.1  	SGLDPISRRKIWDILLAERGSRTIILTTHFLDEADLLADHIAILSKGVLKAKGSSVELKN
          	**:**:****:***:***** **:***************:**::***.*:*:*******:

MG00937.1 	RLGGGYRIHFLKTKTLQDGPV--VEGVTKKVSFDIISYIAPSSALAAEVIRTLEAHDIRD
NCU04021.1	RMGG-YRIHLNNAKYISNSPN--VSGVQKKVSPEEITYIASTSAAAAQVIKELAVAGISD
FG08373.1 	RMGGGYRVHVPKDIGVRETPD--IDGVVKKDAFDLITYTAPSSQLAAVVIKNLEAAGVGE
AN8812.1  	QLGSGYRIHVFNVPGSERAVQKRFSSIHKEVHFDETVYTVQNSAEAAQFVSELEQEGMME
          	::*..**:*. :         . ...: *:   :   * . .*  ** .:  *   .: :

MG00937.1 	YRFSGPTIEDVFLQLAEEVKGETDAASRGSHLASASLQGAN--AEKTYSSEMVT----PD
NCU04021.1	YRFSGPTIEDVFLSLAEEVR-AEDGARLDQFQSTAAVLGAQRSTEKQSSTEMVNNAPVDD
FG08373.1 	YRFSGPTIEDVFLQVAEEIKDEEAFRNNNQALSVPS-------TEKMSSKENSIE-----
AN8812.1  	YRVSGPTIEDVFLKVASELDFEPSRGRVREGERAAD-------HSSEADDENIE------
          	**.**********.:*.*:          .    .         ..  . *         

MG00937.1 	ADKDNLELMSGNKVGIPRQALVLFRKRCTVFKHNWFPSFAAFIIPILAAGLCTLFIRGQS
NCU04021.1	TTKPSLNLLSGQRIGYFKQGVVLFRKRMTILKRNWFPTLAAFAIPIIAAGLVTLFVMGKD
FG08373.1 	--KPGLELTNGQRIGYAKQAMILFRKRLTILKRNRLLYLFAFLLPIFAAGLTAIFVMDET
AN8812.1  	--RRSLHLMTGCRIGIILQAWYLFRKRLTILRRNPLPYLAAFMIPVIAAGLVTLFLQGVA
          	  : .*.* .* ::*   *.  ***** *::::* :  : ** :*::**** ::*: .  

MG00937.1 	PAGCSTQETR----SGQDIFSNDDNDGNSLLLLYGPTSPTITAALNRTIALIRNGPGDST
NCU04021.1	PVGCSPADQ-----SSRRVATNLFASDFDLFMIAGPSDKFSANNLARLFAPIYMATQDNA
FG08373.1 	TPSCQPADQASRTTSRQDIFSQR-EAGEQIYFLMGPTEKFIASGFSNLSSALFPGIPEDN
AN8812.1  	KPGCSGEDS------YRTPESTIADQWDNLLLVIGPSDKVQPELLESFVVSMNDQSDAVQ
          	  .*.  :        :   :       .: :: **:.   .  :      :        

MG00937.1 	GSSAGSPNGGQ-SSSLFN-IESATSFADFNRGIIDNRKNVTPAGFWLGDTNNPPTLAYTS
NCU04021.1	TSSGNSPGIGMNPLDLFKNITLVDTVDEFNSAVVQYRKNITPAAVWLGEDNSVPTMTYRS
FG08373.1 	-------------------VIKVDTFQEFKDSIASGRKIIT-TGFWLGDDKQGPTFAWVA
AN8812.1  	SSSNAS------------QFYIVDDYPDFSDYIKANYSKVTPGGVYLGDSSWQPTIAWKG
          	 ::  :            ..  .    :*.  :    . :*. ..:**: .  **::: .

MG00937.1 	-VPEMQNAFFGQNLMNMLLTNTTLSAKYAAFDTPWQDNTGNSLQLLVYICLALSAYPAFF
NCU04021.1	DSVEMFTAWFGQWITDMMLSNTTIASQFVNFDIPFAPDTGKSLQMLVYMSLALCAYPGFF
FG08373.1 	-NLFISSPIQAQQILDVLLTKTIISTTWSNLEIPISPSTGDSLQMIVYMSIALSVYPAFF
AN8812.1  	DNGNFPLAALTQNVLDRFATGMSINIGFDFFDIPSTPDFYNTLQLVVYFGLAMSVYPAFF
          	.   :  .   * : : : :   :   :  :: *   .  .:**::**: :*:..**.**

MG00937.1 	ALYPNVERRRFVRGLQYSNGVRQFPLWIAYVLFDFINVVISSAIVIGLFVAIADVWYHAG
NCU04021.1	ALYPNVERRSFVRGLQYSNGVRPLPLWLAYTTFDFLIVLVASAIMTALLAGLASVWHHVG
FG08373.1 	ALYPSNERRKYVRGLQYSNGVRPFPLWIAYLLFDLIIVFLATSIAAAIWVGVSDIWFHLG
AN8812.1  	ALYPTVERLRNVRALQFSNGVRALSLWLAYISFDFCLVVASSVLAVIIFRAVTNIWYHIE
          	****. **   **.**:***** :.**:**  **:  *. :: :   :  .::.:*.*  

MG00937.1 	YLFLILVLYGLCAILLSYNISIFASNQLSAYAFAAGFNAVTFLVYLIGYMATITFARVDR
NCU04021.1	YLFIIFVLYGLASILLAYVISLWSNNQLSAYAFTAGGQVVMFLIYLIAYMCTITYGPVDR
FG08373.1 	YVFLILFLYGIASTLFAYIVSLFTSTQLGTFAWAAASQTLVFVVYIVAYMSVLTYTPVTK
AN8812.1  	YLFAVFFLYGLCGTLCAYLVSLFTKSQLAAFAFAAGFQCVMFLIYLIAYMCVLTYASTDK
          	*:* ::.***:.. * :* :*:::..**.::*::*. : : *::*::.**..:*:  . :

MG00937.1 	VDSSLLVVNFVVSAFAPIGSAVRGFFIALNLFSTTCQDQE---LAPNPGGFLQYGSPIMY
NCU04021.1	VDDMLVVVHFVVAIFAPIGSLTRALFIALNLFSTACDGDK---LASYPGGILQYGGPILY
FG08373.1 	VDSTLRLVTFLISAFAPIGSALKALFIATNLFSTACDDTE---LTANPSGILYYGGPILY
AN8812.1  	IDSYIDITHYTIAIVSPSGNLLRALFASLNVFSILCRGSEGREIASYPGEIGLYGGPILY
          	:*. : :. : :: .:* *.  :.:* : *:**  * . :. .::. *. :  **.**:*

MG00937.1 	LIIQAILLFLLLLWLDSGTIRATWQRLFGRKNKNAN----QQAADISDEEVAGELVRVTS
NCU04021.1	LILQSIILFILLVWVDAGNPCSTLKQLFKRKPRSAPPTVAQEADGISDEEVAHELVRVKS
FG08373.1 	LILQSIIFFGLLIWLDTGSAGASIRGLLHGKKKDTD-----SDEEQIDEDIISERNKVTK
AN8812.1  	LILQSIFLVVLLVWIEGGTPLLSWLRPKSRQRDVE-------EKELMDSDIAEEITRVSS
          	**:*:*::. **:*:: *.   :       :                *.::  *  :*..

MG00937.1 	SATDPSRRRQDTKGADGVSSSDKDGLQVVHVTKTFGKNTAVDNVTFGVKHGEVFALLGPN
NCU04021.1	TAT----------GSGVAGTNNTDGLQVVNLTKTFGRNTAVDNVTFGIPHGQVFALLGPN
FG08373.1 	NAE------------------NEHGLRVMHLTKSFRNNTAVDNVTFGIRRGEVFALLGPN
AN8812.1  	SKD---------------------NLRLLHVSKAFKKFIAVEDVTFGVGAGEVFALLGPN
          	.                       .*::::::*:* .  **::****:  *:********

MG00937.1 	GAGKSTSLNMIRGDLQP--SPGGDVFVEGVSVSKQLAAARANLGVCPQYDAIDVMTVTEH
NCU04021.1	GAGKSTTISIIRGDVKPDYNSGGDVFVEQSSVARSLAAARSHLGVCPQFDAVDTMTVLEH
FG08373.1 	GAGKSTTISLIRGDIKPD-RNGGDVLVEDISVNKNLAAARANLGVCPQFDAMDQMTVREH
AN8812.1  	GAGKTTTISLIRGDIQPT-RNEGEIFVENISVLKQRAVARSRLGVCPQFDAMDQMTVLEH
          	****:*::.:****::*     *:::**  ** :. *.**:.******:**:* *** **

MG00937.1 	LRFYARVRGIAD--VDRQVEAVIRAVGLELFRDRQAFALSGGNKRKLSLGIALMGNPSVI
NCU04021.1	LRFYARVRGIPASGIEYNVEQVIRAVGLTQFRDRMARALSGGNKRKLSLGIALMGNPTVV
FG08373.1 	LEFYAKVRGIEE--VEHNVRAVMQAVGLESFATRMAHALSGGNKRKLSLGIALMGNPTVV
AN8812.1  	LVFYARIRGVPD--INHNVNEVINAVGLKQFRHRMAAKLSGGNKRKLSLGIALMGNPSVL
          	* ***::**:    :: :*. *:.****  *  * *  *******************:*:

MG00937.1 	LLDEPSSGLDAAAKRIMWRTLAGTVPGRSILLTTHSMEEADALAGRVGILAKRM1LAMGS
NCU04021.1	LLDEPSSGLDAAAKRIMWRTLAETVPGRSILLTTHSMEEADALAGRAGILAKRM~LAMGS
FG08373.1 	LLDEPSSGLDAASKRVMWKTLEATVPGRSILLTTHSMEEADALAGRAGILARRM~LALGT
AN8812.1  	LLDEPSSGMDAASKRVMWKTLTAVAPGRSIVLTTHSMEEADALAHRAGIMARRM~LALGT
          	********:***:**:**:**  ..*****:************* *.**:*:** **:*:

MG00937.1 	ADNLRHRFGDLLHVHIVLKGAPRTSDADAERVRNWIVQTLP-GAEVEEKVYHGQLRFSVP
NCU04021.1	VDSLRKRFGDTLHVHLVCRGAPRTSDARIAQIHAWVESTFGSAAAMEGKTYHGQMRFSVP
FG08373.1 	PDNLRHRFGDALHIHLVSSTAPRTNDEEMARITDWIRQTLP-SADVDEKTYHGQIRFSVR
AN8812.1  	TDALRLKYGNMYHVHIVHTQAPHTSDEDMEKIRGWVTDNFP-GAVIEQKTYHGQLRFSVP
          	 * ** ::*:  *:*:*   **:*.*    ::  *: ..:  .* :: *.****:**** 

MG00937.1 	ASVVS----GQKQ-RTTEGGEGDEITREGQAAASSRS--------------------SQS
NCU04021.1	AAVVL----SLTTGIINQRADKGMAAEEIRETETQQS--------------------TTS
FG08373.1 	AGQVL----AATS----------GRSEEEITSKDADL--------------------SQS
AN8812.1  	AGISPEKERAAHSDDSKGSSYRDIRSTDELRSVSLRSDVSIPVGLGPDTETGPGNFRARS
          	*.   ... .   .  .  .  .  : :   :     . : . . ...:.:....  : *

MG00937.1 	AVGKLVVMLEENK-HLGIEHYAVSPTTLDQVFLTIVGKHNVKEEG~YNEDEKPTGWKRLF
NCU04021.1	AIGKLVVLLEEQREELGVEHFSVSPTTLDQVFLEVVGRHNVREEG2M-------------
FG08373.1 	AIGHLVVLLEENKQKLGVGHYSVSPTTLDQVFLSIVGQHNVKEEN~SEEKTVSIWRKIWM
AN8812.1  	GVSKLFSQLEQNKAALGVEHYSVSQTTLDQVFLTIVGKHHISEQD~SG------------
          	.:.:*.  **:::  **: *::** ******** :**:*:: *:.               

MG00937.1 	SRRK~-------------
NCU04021.1	----~-------------
FG08373.1 	FGRS2YVVRAFLKWRHAT
AN8812.1  	----~-------------
          	
```
